# TNF Induces Laminin-332-Encoding Genes in Endothelial Cells and Laminin-332 Promotes an Atherogenic Endothelial Phenotype

**DOI:** 10.3390/ijms25168699

**Published:** 2024-08-09

**Authors:** Assim Hayderi, Mulugeta Melkie Zegeye, Sare Meydan, Allan Sirsjö, Ashok Kumar Kumawat, Liza U. Ljungberg

**Affiliations:** Cardiovascular Research Centre, Department of Medical Sciences, School of Medicine, Örebro University, 70362 Örebro, Sweden; assim.hayderi@oru.se (A.H.); jaje93@hotmail.com (S.M.); allan.sirsjo@oru.se (A.S.); ashok.kumawat@oru.se (A.K.K.)

**Keywords:** laminin-5, ECM, activated endothelial cells, monocytes migration, atherosclerosis

## Abstract

Laminins are essential components of the basement membranes, expressed in a tissue- and cell-specific manner under physiological conditions. During inflammatory circumstances, such as atherosclerosis, alterations in laminin composition within vessels have been observed. Our study aimed to assess the influence of tumor necrosis factor-alpha (TNF), a proinflammatory cytokine abundantly found in atherosclerotic lesions, on endothelial laminin gene expression and the effects of laminin-332 (LN332) on endothelial cells’ behavior. We also evaluated the expression of LN332-encoding genes in human carotid atherosclerotic plaques. Our findings demonstrate that TNF induces upregulation of LAMB3 and LAMC2, which, along with LAMA3, encode the LN332 isoform. Endothelial cells cultured on recombinant LN332 exhibit decreased claudin-5 expression and display a loosely connected phenotype, with an elevated expression of chemokines and leukocyte adhesion molecules, enhancing their attractiveness and adhesion to leukocytes in vitro. Furthermore, LAMB3 and LAMC2 are upregulated in human carotid plaques and show a positive correlation with TNF expression. In summary, TNF stimulates the expression of LN332-encoding genes in human endothelial cells and LN332 promotes an endothelial phenotype characterized by compromised junctional integrity and increased leukocyte interaction. These findings highlight the importance of basement membrane proteins for endothelial integrity and the potential role of LN332 in atherosclerosis.

## 1. Introduction

**Figure 1:**
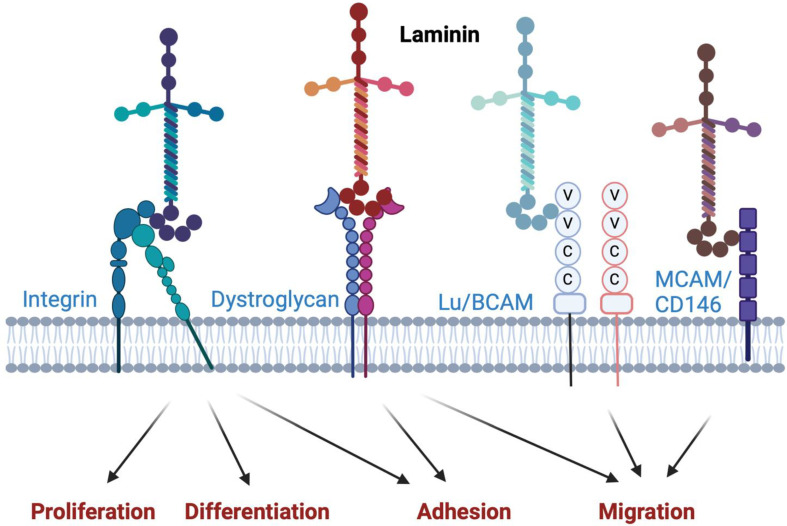
Laminin–receptor interactions and their consequences. Schematic figure illustrating the interaction between laminins and their receptors, such as integrins, dystroglycans, Lutheran/basal cell adhesion molecule (Lu/BCAM), and melanoma cell adhesion molecule (MCAM/CD146), and the consequences of these interactions on the behavior and phenotype of the cells. Created with BioRender.com.

Laminins are heterotrimeric glycoproteins and key components of the basement membranes, where they function as scaffold proteins to anchor the surrounding cells to the extracellular matrix [1]. In addition, laminins serve as large signaling molecules by interacting with cell surface receptors such as integrins, dystroglycans, Lutheran/basal cell adhesion molecule (Lu/BCAM), and melanoma cell adhesion molecule (MCAM/CD146), thereby influencing the behavior and phenotype of the cells (Figure 1) [1,2]. Laminins consist of an alpha, a beta, and a gamma chain [1,2]. Under physiological conditions, these chains exhibit tissue- and cell-specific expression, with laminin-211, -411, -421, -511, and -521 being considered vascular laminins [2,3,4,5]. However, the expression of laminin genes has been reported to be altered in aged tissues and in atherosclerotic arteries [6,7,8]. Proinflammatory cytokines such as interleukins, interferon gamma, and tumor necrosis factor-alpha (TNF), all abundantly expressed in atherosclerotic lesions, have been reported to affect the expression of laminin genes in various cell types, including vascular endothelial cells [9,10].

Endothelial cells, in their intact and quiescent state, are essential for maintaining vascular homeostasis [11]. However, when activated, these cells play a crucial role in the initiation and progression of atherosclerosis [12]. The activation of endothelial cells results in reduced junctional integrity and in an increased expression and secretion of adhesion molecules and chemokines [13,14]. These changes facilitate the recruitment, adhesion, and extravasation of leukocytes into the arterial wall, triggering the further expression and secretion of inflammatory cytokines, thereby creating a cycle of persistent chronic inflammation.

**Figure 2:**
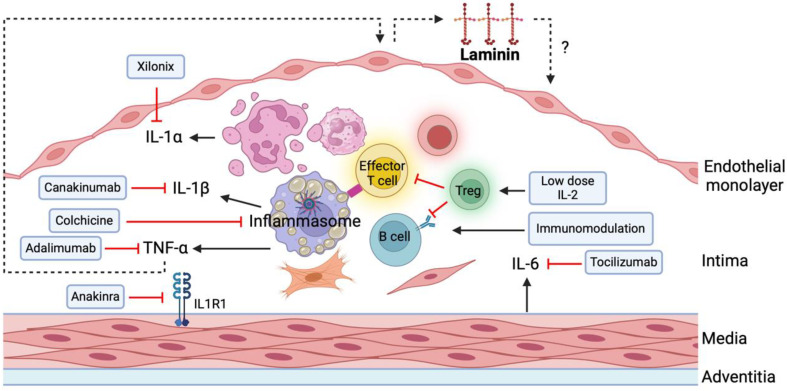
Major cells and cytokines of atherosclerotic plaques. A schematic figure illustrating the key cellular components and cytokines present within atherosclerotic plaques, including macrophages, vascular smooth muscle cells (VSMCs), and T cells. These cells contribute to the progression of atherosclerosis through various mechanisms. Upon the uptake of modified low-density lipoproteins (LDL), these cells secrete proinflammatory cytokines that act on different cell types, thereby accelerating the disease process. Among these cytokines, TNF is a potent activator of endothelial cells and plays a crucial role in modulating laminin gene expression. The figure also highlights potential inhibitors of these cytokines, offering insight into therapeutic strategies aimed at mitigating atherosclerotic progression. Created with BioRender.com.

TNF is a key driver of inflammation in atherosclerosis, targeting the expression of numerous inflammatory genes in different cells of the vessel wall, including the endothelial cells (Figure 2) [15]. However, its impact on the expression and turnover of endothelial extracellular matrix proteins remains elusive. Investigating whether inflammatory stimuli like TNF affect extracellular matrix gene expression and elucidating the consequences of such changes for vascular integrity may provide further understanding of the molecular mechanisms driving atherosclerosis development.

To address this knowledge gap, we aimed to investigate the impact of TNF on laminin gene expression in cultured human endothelial cells. Specifically, we wanted to explore whether TNF promotes the expression of genes that give rise to certain laminin isoforms in order to evaluate the effect of these laminin isoforms on endothelial cells’ behavior and function. Additionally, we utilized human carotid atherosclerotic data to examine the expression and correlation of laminin genes with TNF within atherosclerotic lesions.

Our study demonstrates that TNF acts as a strong inducer of genes encoding the laminin-332 (LN332) isoform and reveals new knowledge of how TNF indirectly affects endothelial function. Further, we show that LN332 upon interaction with endothelial cells promote an atherogenic endothelial phenotype, characterized by high expression levels of chemokines and adhesion molecules and low expression levels of the endothelial tight junction protein, claudin-5. The cell culture supernatant from these cells is more attractive to monocytes, and these cells exhibit increased adhesion to leukocytes in vitro. Consistent with these findings, we observed significantly higher levels of LN332-encoding genes in human carotid lesions, which also displayed a significant correlation with TNF levels.

## 2. Results

### 2.1. TNF Alters Laminin Gene Expression in HUVECs

**Figure 3:**
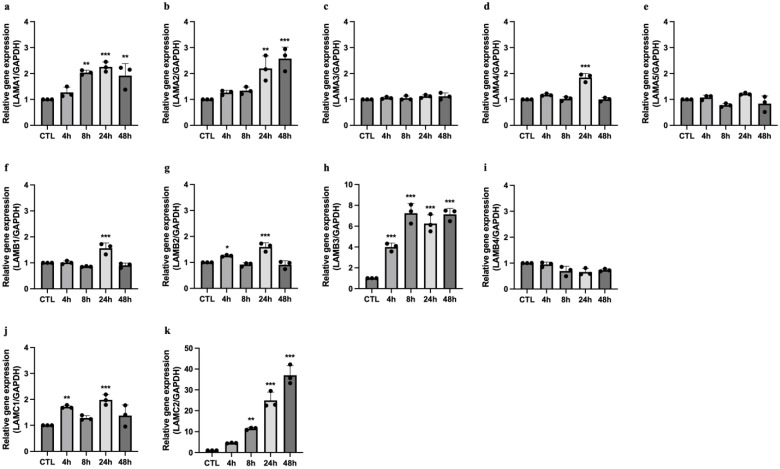
TNF alters the mRNA expression of laminin-encoding genes in human endothelial cells. The mRNA expression of *LAMA1–5* (**a**–**e**), *LAMB1–4* (**f**–**i**), and *LAMC1* and *LAMC2* (**j**,**k**) in human endothelial cells exposed to 50 ng/mL of TNF-α for 4–48 h. The data are presented as mean ± SD of 3 independent experiments. One-way ANOVA followed by Dunnett’s multiple comparison were performed to calculate statistical significance. All the timepoints are compared to CTL. * *p*-value < 0.05, ** *p*-value < 0.01, *** *p*-value < 0.001.

To investigate the effects of TNF on the expression of laminin-encoding genes, we quantified the transcripts of the five α, four β, and three γ laminin genes in human umbilical vein endothelial cells (HUVECs) treated with TNF for 4–48 h. Among the laminin α genes, *LAMA1*, *LAMA2*, and *LAMA4* transcripts were significantly upregulated by TNF in at least one of the tested timepoints (Figure 3a,b,d). Among the four β genes, *LAMB1*, *LAMB2*, and *LAMB3* mRNAs were significantly upregulated, while *LAMB4* was downregulated by TNF (Figure 3f–i). Furthermore, we observed a significant increase in the mRNA expression of both *LAMC1* and LAMC2 by TNF, whereas *LAMC3* mRNA was not detected (Figure 3j,k).

### 2.2. TNF Induces the Expression of Laminin Genes That Make up the LN332 Isoform

**Figure 4:**
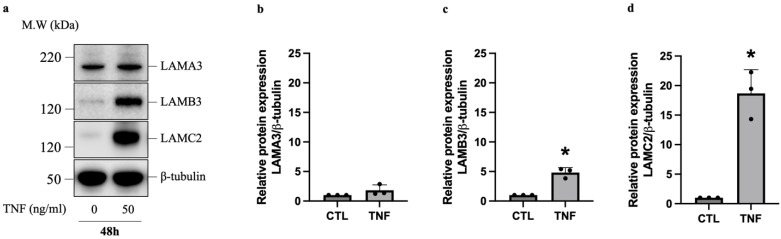
TNF alters the protein expression of LN332-encoding genes in human endothelial cells. Protein expression of LN332-encoding genes in HUVECs following treatment with TNF for 48 h (**a**–**d**). Figure (**a**) shows representative cropped Western blot image of LN332 chains, while Figures (**b**–**d**) show relative levels of LN332 chains from three independent experiments (CTL = 1 a.u.). Full-length Western blot images are shown in Supplementary Figure S1. The data are presented as mean ± SD. Student’s *t*-test was conducted to calculate statistical significance. * *p*-value < 0.05.

Gene expression analysis revealed that TNF induces a dramatic upregulation of LAMB3 and LAMC2, which, along with LAMA3, encode the LN332 isoform. To determine if the increased mRNA expression of LN332-encoding genes correlates with increased protein expression following TNF treatment, we quantified the protein levels of LAMA3, LAMB3, and LAMC2 in HUVECs stimulated with TNF for 24–72 h using Western blot. Our results show that untreated HUVECs exhibit a moderate basal expression of LAMA3 and low basal expression of LAMB3 and LAMC2 (Figure 4a). A pronounced increase in the protein levels of both LAMB3 and LAMC2 was observed upon TNF treatment of HUVECs for 48 h (Figure 4a,c,d). Although LAMA3 protein level was, on average, increased by 83%, it was not statistically significant (*p* = 0.255) (Figure 4b). Moreover, we conducted mRNA and protein analyses of these chains in human aortic endothelial cells (HAECs) following a 48 h treatment with TNF to determine if similar results could be observed in a different type of endothelial cells, thereby assessing the reproducibility of our findings beyond HUVECs. In contrast to HUVECs, HAECs treated with TNF displayed a statistically significant increase in the mRNA levels of all three chains (Figure S2a–c). However, we only observed a significant increase in the protein expression of LAMC2 upon TNF treatment in HAECs (Figure S3a–g).

### 2.3. HUVECs Cultured on LN332 Display Altered Morphology and Compromised Integrity

**Figure 5:**
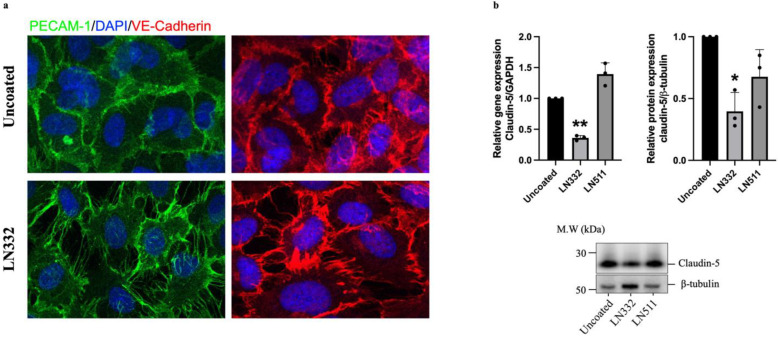
Human endothelial cells cultured on LN332 display irregular shape, appears loosely connected and express less tight junction protein claudin-5. A representative PECAM-1 and VE-cadherin staining of human endothelial cells cultured on uncoated or LN332-coated surfaces for 48 h (**a**). mRNA and protein expression of claudin-5 in human HUVECs cultured on plastic, LN332, or LN511 for 48 h (**b**). The data are presented as mean ± SD of three independent experiments. One-way ANOVA and Bonferoni test were performed to assess statistical significance. Cells cultured on laminins are compared to cells cultured on plastic (uncoated). * *p*-value < 0.05, ** *p*-value < 0.01.

Next, we decided to investigate whether endothelial cells respond to LN332 by culturing HUVECs on uncoated or LN332-coated surfaces and comparing their morphological parameters. We observed a clear difference between cells cultured on LN332 for 48 h and those cultured on uncoated surfaces. These cells were stained using antibodies targeting platelet-endothelial cell adhesion molecule (PECAM)-1 and vascular endothelial (VE)-cadherin. The staining shows that cells cultured on LN332 exhibit an irregular shape and appears loosely connected, indicating a distinct response of endothelial cells to LN332 (Figure 5a). In addition, gene and protein expression analysis revealed a significantly lower expression of claudin-5, an endothelial tight junction protein, at both mRNA and protein levels in cells cultured on LN332 compared to cells cultured on uncoated or LN511-coated surfaces (Figure 5b and Figure S4). Consistent with these findings, we observed a significant reduction in claudin-5 mRNA in HAECs cultured on LN332 for 48 h (Figure S5a).

### 2.4. LN332 Induces the Expression and Secretion of Leukocyte Adhesion Molecules

**Figure 6:**
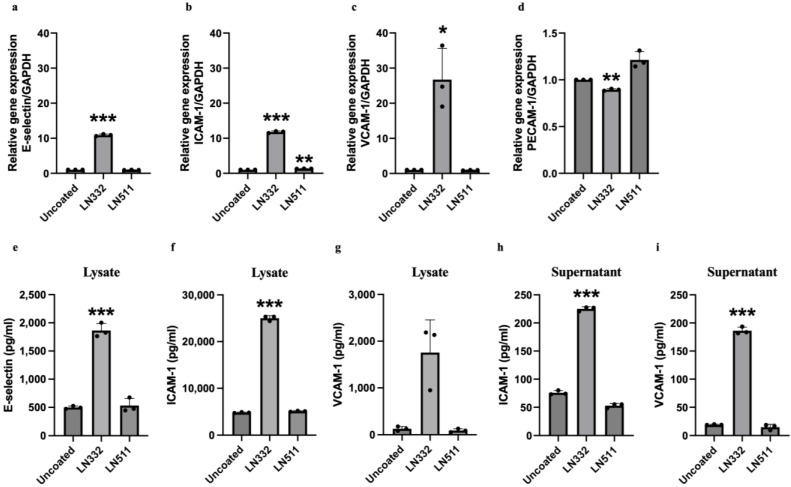
Endothelial cells cultured on LN332 have higher expression and secretion of leukocyte adhesion molecules. mRNA expression of *E-selectin* (**a**), *ICAM-1* (**b**), *VCAM-1* (**c**), and *PECAM-1* (**d**) in human endothelial cells cultured on uncoated plastic, LN332, or the normal vascular laminin isoform, LN511, for 48 h. Protein levels of E-selectin (**e**), ICAM-1 (**f**), and VCAM-1 (**g**) in cell lysate and protein levels of ICAM-1 (**h**) and VCAM-1 (**i**) in supernatant of endothelial cells cultured on plastic, LN332, or LN511 for 48 h. The data are presented as mean ± SD of three independent experiments. One-way ANOVA and Bonferroni test were performed to calculate statistical significance. * *p*-value < 0.05, ** *p*-value < 0.01 and *** *p*-value < 0.001 comparing cells cultured on laminins to cells cultured on uncoated plastic.

Endothelial cells with disrupted junctional integrity typically exhibit an enhanced inflammatory response [16]. Inflammatory endothelial cells tend to be sticky and secrete chemoattractants to facilitate the recruitment, adhesion, and extravasation of leukocytes [16]. In light of this, we decided to expand our investigation to evaluate various adhesion molecules, comprising endothelial *(E)-selectin*, intracellular cell adhesion molecule *(ICAM)-1*, vascular cell adhesion molecule *(VCAM)-1*, and platelet-endothelial cell adhesion molecule *(PECAM)-1*, which are considered markers of endothelial cells undergoing activation [12,13,14]. While *PECAM-1* mRNA was slightly but significantly downregulated, we observed a significantly higher mRNA expression of *E-selectin*, *ICAM-1,* and *VCAM-1* in cells cultured on LN332 in comparison to cells cultured on an uncoated surface (Figure 6a–d), and we also observed a clear increase in the protein levels of all three adhesion molecules in the lysate of cells cultured on LN332 (Figure 6e–g). Cells cultured on the normal vascular laminin isoform, LN511, displayed a slightly higher *ICAM-1* mRNA compared to cells cultured on an uncoated surface, but this increase was not seen on the protein level (Figure 6b,f). A clear increase in the protein levels of all three adhesion molecules was evident in the lysate of cells cultured on LN332 as compared to lysate from cells cultured on uncoated or LN511-coated surfaces (Figure 6e–g). In addition, increased levels of soluble ICAM-1 and VCAM-1 were found in the supernatant of cells cultured on LN332 (Figure 6h,i), while *E-selectin* could not be detected in the supernatant. Analysis of *E-selectin* mRNA expression in HAECs cultured on LN332 for 48 h showed similar results (Figure S5c). Together, these findings indicate that LN332 promotes activation of endothelial cells to favor the recruitment and adhesion of leukocytes to endothelial cells.

### 2.5. HUVECs Cultured on LN332 Exhibit Increased Chemokine Secretion

**Figure 7:**
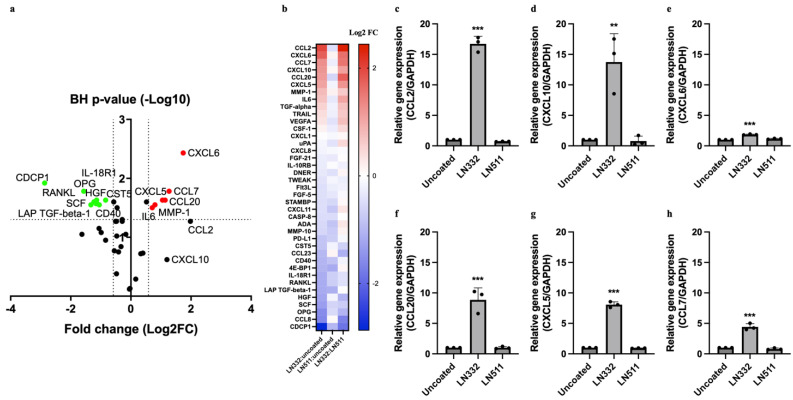
Human endothelial cells cultured on LN332 have higher expression and secretion of chemokines. Volcano plot showing significantly up- and downregulated proteins in supernatant of endothelial cells cultured on LN332 in relation to cells cultured on uncoated surface for 48 h as measured by OLINK’s proximity extension assay (**a**). Red-colored dots indicate proteins that are significantly upregulated, and green-colored dots indicate proteins that are significantly downregulated (Log2FC < 0.58 and false discovery rate 5%). Heatmap showing comparison of proteins detected in supernatant from cells cultured on LN332 with cells cultured on uncoated or LN511-coated surfaces (**b**). Gene expression of upregulated chemokines in human endothelial cells cultured on plastic, LN332, or LN511 for 48 h determined by qRT-PCR (**c**–**h**). OLINK data are presented as mean log2 fold change of four independent experiments. qRT-PCR data are presented as mean ± SD of three independent experiments. One-way ANOVA and Bonferroni test were performed to calculate statistical significance for PCR data, whereas *t*-test and Benjamini–Hochberg tests were used to evaluate statistical significance for OLINK data. Cells cultured on laminins are compared to cells cultured on plastic (uncoated). ** *p*-value < 0.01, *** *p*-value < 0.001.

Next, we used the OLINK platform to assess the release of inflammatory proteins from HUVECs cultured on LN332, LN511, or an uncoated surface for 48 h. The supernatants were analyzed using OLINK’s target 96 inflammation panel, which detects 92 unique proteins. Of these proteins, 39 were above the assay’s detection limit. The analysis of these 39 proteins showed that HUVECs cultured on LN332 exhibited distinct patterns of inflammatory protein release compared to those cultured on LN511 or an uncoated surface. Specifically, the levels of CXCL6, CCL7, CCL20, CXCL5, MMP1, IL6, CCL2, and CXCL10 were significantly higher in the supernatant of cells cultured on LN332 compared to cells cultured on an uncoated surface. Meanwhile, CCL2 and CXCL10 were increased but did not reach statistical significance (Figure 5a). In contrast, the levels of CDCP1, OPG, RANKL, SCF, HGF, IL18R1, LAP TGF-β1, CD40, and CST5 were lower in the supernatant of cells cultured on LN332 (Figure 7a). Similar results were seen in supernatant from cells grown on LN332 compared to LN511-coated surfaces (Figure 7b). To validate these findings, we further evaluated the mRNA expression of several of the proteins upregulated by LN332. In line with the OLINK results, the mRNA levels of these genes were significantly elevated in cells cultured on LN332 (Figure 7c–h) suggesting that LN332 may affect the expression of these genes on transcriptional level. Moreover, we examined the mRNA expression of MCP-1 in HAECs cultured on LN332 for 48 h to assess reproducibility in a different endothelial cell type and the results were consistent with those obtained from HUVECs (Figure S5b).

**Figure 8:**
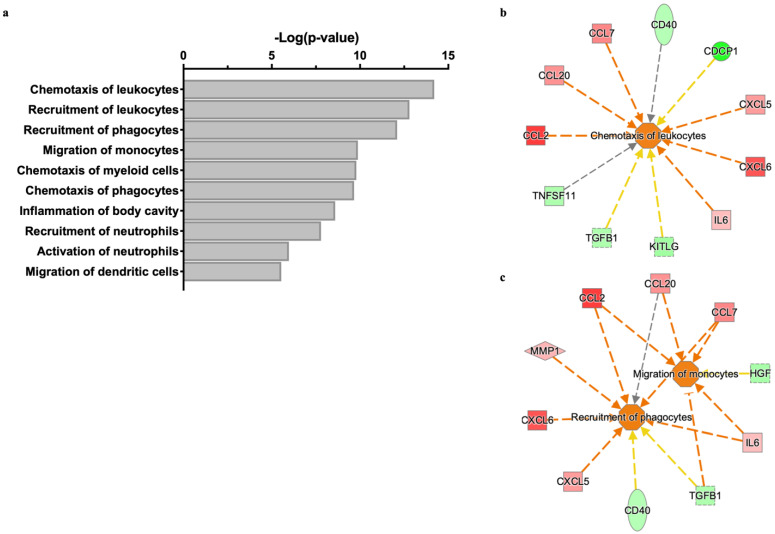
Enrichment analysis predicts that human endothelial cells cultured on LN332 release proteins that regulate leucocyte migration/chemotaxis. Ingenuity Pathway Analysis (IPA) was used to perform enrichment analysis of differentially regulated proteins (Log2FC ± 0.58 and false discovery rate 20%) released from human endothelial cells cultured on LN332 compared to cells cultured on plastic. A bar graph showing the top ten functions (Z-score > 1.5) enriched by LN332 regulated proteins with their respective *p*-values (−Log10) (**a**). Proteins that enriched the functions “chemotaxis of leucocytes” (**b**) “and recruitment of phagocytes” or “migration of monocytes” (**c**) are presented with their respective predicted impacts on the activation state of the functions. The red color indicates upregulation in release of proteins while green indicates downregulation. Orange lines indicate that a protein leads to predicted activation of function, while yellow lines show disagreement between the state of differentially regulated protein expression and the predicted sate of function. The gray lines indicate that no prediction could be made.

Using the cutoff values of ±1.5 fold-change in protein level and FDR-value of 20%, we extracted eight downregulated and six upregulated proteins from our OLINK data. These proteins were evaluated using Ingenuity Pathway Analysis (IPA) software (IPA^®^, QIAGEN Inc., Hilden, Germany. https://www.qiagenbioinformatics.com/products/ingenuity-pathway-analysis, analysis date 14 March 2022) for enriched biological functions. Among the top enriched biological functions, we observed functions related to chemotaxis/migration of leukocytes to be activated (Figure 8a). The proteins that enriched the top three biological functions are presented with their respective predictive impacts on the activation state of the functions (Figure 8b,c).

### 2.6. HUVECs Cultured on LN332 Facilitate Migration of Monocytes and Adhesion of PBMCs In Vitro

**Figure 9:**
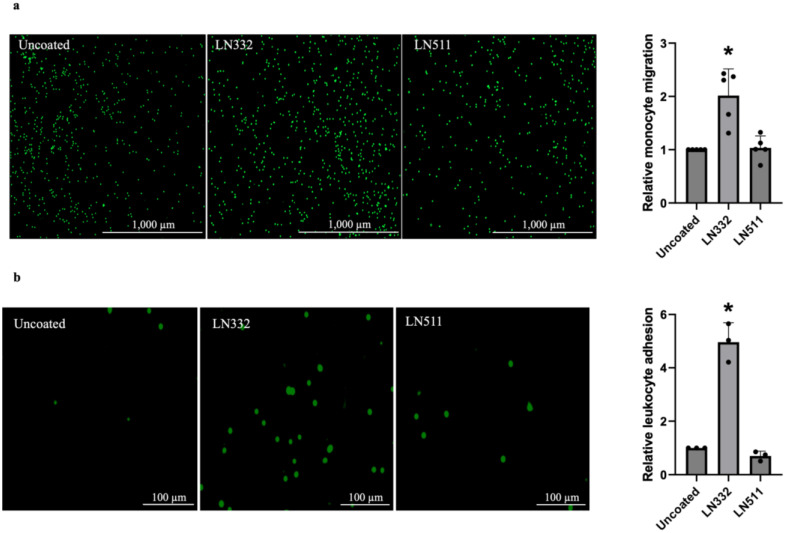
Monocytes tend to migrate more towards supernatant from endothelial cells cultured on LN332 and leukocytes adhere more to endothelial cells cultured on LN332. In vitro migration of CD14^+^ monocytes towards the supernatant from endothelial cells cultured on plastic, LN332, or LN511 performed in Boyden’s transwell system (**a**) (n = 5). Adhesion of leukocytes to endothelial cells cultured on plastic, LN332, or LN511 (**b**) (n = 3). The data are presented as mean ± SD. One-way ANOVA followed by Bonferroni test were performed to calculate statistical significance. * *p*-value < 0.05 comparing cells cultured on laminins to cells cultured on uncoated plastic.

To investigate the functional implications of these findings, we assessed the adhesive properties of endothelial cells cultured on LN332 and the migratory response of primary monocytes towards the conditioned medium from cells cultured on LN332. Using Boyden’s transwell assay, we observed that the conditioned medium from cells cultured on LN332 exhibited a higher ability to attract human CD14^+^ monocytes compared to conditioned medium from cells cultured on an uncoated surface or LN511 (Figure 9a). Moreover, the adhesion of PBMCs to endothelial cells cultured on LN332 was greatly increased compared to cells cultured on uncoated or LN511-coated surfaces (Figure 9b). Notably, this effect was not attributed to the direct binding of PBMCs to the laminins, as leukocytes showed a higher preference for binding to LN511 over an uncoated surface or LN332 in the absence of endothelial cells (Figure S6). These findings suggest that LN332 may play an important role in activation of endothelial cells to promote recruitment and adhesion of leukocytes.

### 2.7. LN332-Encoding Genes are Elevated in Carotid Atherosclerotic Lesions and Correlate with TNF

**Figure 10:**
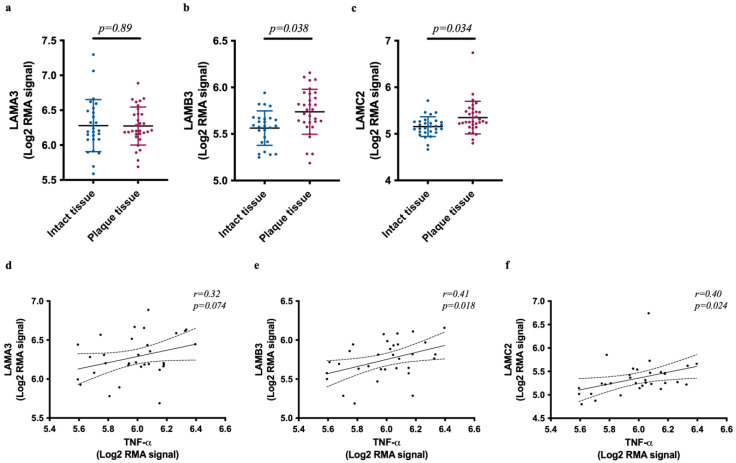
LN332-encoding genes’ transcripts are elevated and correlate with TNF in human carotid atherosclerotic lesions. Gene expression of *LAMA3* (**a**), *LAMB3* (**b**), and *LAMC2* (**c**) in human carotid atherosclerotic tissues and adjacent macroscopically intact tissues (n = 32). Pearson’s correlation of *LAMA3* (**d**), *LAMB3* (**e**), and *LAMC2* (**f**) with *TNF* in human carotid atherosclerotic lesions (n = 32). Solid line indicates Pearson’s correlation coefficient (r), and dashed line indicates 95% confidence band of the best-fit line. Data are acquired from human carotid atheroma gene expression (accession number, GSE43292). *p*-value smaller than 0.05 is considered statistically significant.

Finally, to investigate the expression of genes encoding LN332 in human carotid atherosclerotic tissues and their correlation with TNF, we utilized the genome-wide expression study of human carotid atheroma (GEO accession: GSE43292). Comparison of the expression of these genes in diseased and proximal adjacent macroscopically intact tissues revealed a significantly higher mRNA expression of *LAMB3* and *LAMC2*, but not *LAMA3*, in diseased tissues (Figure 10a–c). Furthermore, both LAMB3 and LAMC2 display a significant correlation with TNF in diseased tissues (Figure 10e,f). The correlation between *LAMA3* and *TNF,* on the other hand, was weaker and not statistically significant (Figure 10d).

## 3. Discussion

Here, we demonstrate that endothelial cells cultured under the influence of TNF express epithelial cell-associated LN332-encoding genes. Endothelial cells cultured on LN332 exhibit changes in morphology and express higher levels of leukocyte adhesion molecules and chemokines, which promote the recruitment and adhesion of leukocytes in vitro. Analysis of microarray data from human carotid vessels reveals higher mRNA levels of LN332-encoding genes in lesions, which correlate with TNF expression.

LN332 is highly enriched in epithelial basement membranes, where it is believed to be important for adhesion and differentiation of these cells [17]. HUVECs, a well-established EC model [18], exhibit no or minimal basal expression of LAMB3 and LAMC2, while the basal expression of LAMA3 in these cells seems rather stable [19]. LAMA3, which comes in two variants—a truncated LAMA3A and a full-sized LAMA3B—appears to have different tissue distribution patterns. LAMA3B, which is believed to be a component of laminin-311, laminin-321, and laminin-3B32, is expressed in the vascular basement membrane of normal tissues, probably as laminin-3B11 or laminin-3B21, since vascular ECs do not express LAMB3 or LAMC2 under physiological circumstances [19]. In our study, we found TNF to induce both LAMB3 and LAMC2 in HUVECs, which may indicate that endothelial cells under inflammatory circumstances may also produce LN332 in addition to laminin-311 and laminin-321. These results were also replicated in HAECs, which reinforces the generalizability of our findings across different endothelial cells and is in line with studies from epithelial cells expressing high levels of LAMC2 in response to TNF [20,21].

When culturing endothelial cells on LN332, we observed morphological alterations accompanied by a reduction in the expression of claudin-5, an endothelial tight junction protein that regulates endothelial tissue barrier permeability [22]. These observations suggest the presence of LN332 receptors on endothelial cells and indicate that LN332 may increase endothelial cell permeability. Indeed, the major LN332-binding integrins, α3β1, α6β1, and α6β4, which are abundant on epithelial cells, have also been reported to be expressed on endothelial cells [23,24,25]. Interestingly, α3β1 integrin is also utilized by the classical endothelial laminins, LN411 and LN511 [24]. The mechanism by which LN332 exerts its effects on endothelial cells, and whether this involves a receptor shared with LN511, remains to be elucidated. Should this be the case, it raises the intriguing question of how LN332 induces distinctly different effects compared to the traditional endothelial laminin, LN511. Although, we could not reliably assess the paracellular permeability of endothelial cells cultured on LN332, we observed a clear loss of contact between cells when cultured on LN332. TNF, which induces LN332-encoding genes, has been reported to increase endothelial permeability by delocalizing and disrupting claudin-5 in HUVECs and human dermal microvascular endothelial cells, respectively [26,27]. To what extent TNF-induced LN332 is involved in TNF-induced dysregulation of endothelial permeability and whether it is a direct or indirect effect of LN332 remains to be evaluated. Nonetheless, invasive tumor cells expressing LN332-encoding genes have been postulated to induce vascular permeability to escape the circulation [28,29]. Higher endothelial permeability is a characteristic of atherosclerosis-susceptible arterial sites, which also tend to have reduced expression of claudin-5 [30].

Moreover, cells cultured on LN332 displayed an increased expression and secretion of chemokines, which were predicted by IPA to be important for orchestrated recruitment of leukocytes. More specifically, these chemokines are widely recognized for their vital role in the recruitment of monocytes (CCL2), neutrophils (CXCL5/6), and lymphocytes (CXCL10), which have all been implicated in the pathogenesis of atherosclerosis [31,32,33,34,35]. Notably, a similar expression pattern for these chemokines and adhesion molecules has been observed in HUVECs after stimulation with lipopolysaccharide (LPS) and TNF [36]. Both LPS and TNF are potent activators of the NFκB signaling pathway, which seems to be also weakly activated by LN332 in epithelial tumor cells [37,38]. The increased expression and secretion of these chemokines by LN332 consequently attracted more monocytes in vitro and these cells were more adhesive to leukocytes suggesting that LN332 may facilitate the recruitment of leukocytes to promote inflammation. Interestingly, classical monocytes (LY6C^hi^/CD14^+^ CD16^−^) are also believed to be recruited to tumor microenvironment, where they play a pro-angiogenic role, thereby promoting tumor growth [39]. These monocytes are characterized by high expression of CCR2 [40], the main receptor for CCL2, a chemokine strongly induced by LN332 in endothelial cells. In addition, we observed a significantly higher level of ICAM-1, which has been suggested as an important mediator of CD14^+^ monocyte adhesion in tumor microenvironment [41]. Considering that LAMB3 and LAMC2 are established prognostic markers in various cancer types [42,43], our findings may also have implications for endothelial cells within tumor microenvironment. It is noteworthy that the metabolic similarities between tumor and atherosclerotic plaque microenvironments have only recently begun to be explored [44,45].

A 67-kDa laminin receptor (67LR), which is elevated in metastatic tumors and is also expressed on vascular cells, has emerged as an interesting therapeutic target for both cancer and cardiovascular diseases [46]. Activation of this receptor with its natural ligand, (-)-epigallocatechin-3-O-gallate (EGCG), is associated with an increased production of cGMP, which has been reported to induce death in tumor cells [47]. In VSMCs, the induction of cGMP is associated with the relaxation of these cells, which is a key mechanism in the regulation of blood pressure, which is a risk factor for cardiovascular diseases. This process is typically mediated by the activation of guanylate cyclase, often in response to nitric oxide secreted by endothelial cells. However, whether LN332 specifically interacts with 67LR to induce cGMP production in VSMCs remains, to the best of our knowledge, unknown.

Finally, to extend our findings to human atherosclerotic disease, we evaluated the levels of LN332-encoding genes in diseased and macroscopically intact adjacent carotid tissues (GEO accession number: GSE43292) [48]. The diseased tissues in this dataset have been evaluated by pathologists and classified as stage IV or higher according to Stary’s classification of atherosclerotic plaques, whereas the intact adjacent tissues are classified as stage II or lower [49]. Consequently, the diseased tissues in this dataset exhibit significantly higher levels of macrophage and lymphocyte markers, enhancing the reliability of this dataset for validating of our in vitro findings. Here, we found that both LAMB3 and LAMC2 were significantly upregulated in lesions, and their expression levels correlate with TNF. These findings support our in vitro findings and highlights the relevance of these findings for the pathophysiology of atherosclerosis and cancer.

In conclusion, our findings demonstrate that TNF significantly alters the expression of genes encoding LN332 in human endothelial cells. Culturing endothelial cells on LN332 induces a transition from quiescent to an activated state, which is crucial for the initiation and progression of atherosclerosis. This highlights the potential of LN332 as a key player in vascular inflammation. Targeting the interaction between LN332 and its receptors specifically in endothelial cells, while preserving its essential functions in epithelial tissues, could offer a novel therapeutic strategy to mitigate vascular inflammation and reduce the risk of atherosclerosis.

## 4. Materials and Methods

### 4.1. Cell Culturing and Treatment

Human umbilical vein endothelial cells (HUVECs) (catnr. C01510C, Thermo Fisher Scientific, Waltham, MA, USA) and human aortic endothelial cells (HAECs) (catnr. CC-2535, Lonza, Basel, Switzerland) were cultured in Vasculife (catnr. LM-0002, Lifeline cell technology, Frederick, MD, USA) supplemented with VEFG LifeFactor kit (catnr. LS-1029, Lifeline cell technology) and 10 U/mL penicillin and streptomycin (PEST) (catnr. 15-140-122, Life technologies, Carlsbad, CA, USA) at 37 °C and 5% CO_2_. Medium was changed every 48 h and cells from passage 3 to 9 were sub-cultured or used for experiments upon 80–90% confluency by detachment with 0.05% trypsin–EDTA (catnr. 15-400-054, Life Technologies). For evaluation of laminin gene expression upon stimulation with TNF, 3 × 10^5^ cells/well were seeded in 6-well culture plates and treated the next day with human recombinant TNF (catnr. 10291-TA-050, R&D systems, Minneapolis, MN, USA) at a concentration of 50 ng/mL for 4 to 72 h. For evaluation of endothelial cells’ responses to different laminin isoforms, 24- or 6-well culture plates were precoated with 0.5 μg/cm^2^ laminin-332 (LN332) (catnr. LN332-0502, BioLamina AB, Sundbyberg, Sweden), LN511 (catnr. LN511-0502, BioLamina AB) or left uncoated for 2 h at 37 °C. For 24- and 6-well plates, 6 × 10^4^ and 3 × 10^5^ cells/well, respectively, were seeded and incubated for 48 h at 37 °C and 5% CO_2_.

### 4.2. Quantitative Real Time-PCR

Total RNA was isolated from frozen cells (stored at −80 °C for maximum 2 weeks) using the E.Z.N.A Total RNA Kit (catnr. R6934-02, Omega Bio-Tek, Norcross, GA, USA) following the manufacturer’s protocol. Cells were lysed with TRK lysis buffer containing 2% beta-mercaptoethanol on ice. After adding 70% ethanol, the lysate was transferred to columns and centrifuged. The columns were washed three times with wash buffers, dried, and RNA was eluted with nuclease-free water. RNA quantity and purity were assessed using NanoDrop 2000 Spectrophotometer (Thermo Scientific). These samples were stored at −80 °C.

Complementary DNA (cDNA) was synthesized from 1 μg of total RNA using the High-Capacity cDNA Reverse Transcription Kit (catnr. 43-749-66, Thermo Scientific). The RNA, along with nucleotides, random primers, reverse transcriptase, and buffer, was mixed in PCR tubes and the reaction was run in a Biometra UNO-Thermoblock (Biometra, Göttingen, Germany). These samples were stored at −20 °C.

For quantitative real-time PCR, LuminoCT ReadyMix (catnr. L6669, Sigma-Alcrich, St. Louis, MO, USA) and specific primers/probes (thermo Scientific) were used with the QuantStudio 7 Flex Real-Time PCR system (Thermo Scientific). In brief, 1 μL cDNA was combined with 9 μL master mix in a 96-well PCR plate, which was centrifuged and ran in the QuantStudio 7 Flex Real-Time PCR system (Thermo Scientific) for 40 cycles. Gene expression was normalized to GAPDH expression, and relative gene expression was determined using a 6-point standard curve from serial dilutions of pooled cDNA.

### 4.3. ELISA

Quantification of ICAM-1 (catnr. DY720-05), VCAM-1 (catnr. DY809-05) and E-selectin (catnr. DY724) in supernatant and lysate (stored at −80 °C for up to 1 year) was performed using human Duoset ELISA kits (R&D systems) following manufacturer’s protocol. Briefly, 96-well plates were coated with capture antibody and incubated overnight at room temperature on a shaker. The next day, plates were washed with PBS containing 0.05% Tween-20 (Sigma-Aldrich); then, cell samples and standards were added for a 2 h incubation on a shaker at room temperature. After washing, detection antibody was added, and the plates were incubated on a shaker for 2 h at room temperature. Following another wash, HRP was added, and the plate was incubated for 20 min. After discarding the HRP and washing, substrate (R&D systems) was added and incubated for 20 min. The reaction was stopped with 1N H_2_SO_4_, and the absorbance was measured at 450 nm using Cytation 3 plate reader (BioTek, Winooski, VT, USA).

### 4.4. Western Blot

RIPA buffer (catnr. 20-188, Millipore, Burlington, MA, USA) containing 1% protease inhibitor (catnr. P187786, Thermo Scientific) was used to lyse frozen cells on ice. BCA Protein Assay Kit (catnr. PI23227, Thermo Scientific) was used as per supplier’s protocol to quantify the total protein concentration. The cell lysate (stored at −20 °C) was denatured using 5× SDS at 95 °C for 5 min. A total of 15 μg protein from each sample was loaded on to 4–12% NuPage Novex Bis-Tris gels (catnr. NP0323BOX, Thermo Scientific) and the proteins were separated using MOPS running buffer (catnr. NP0001, Life Technologies) at 140 V for 95 min. The separated proteins were then transferred to methanol-activated Immobilon-FL PVDF membrane (catnr. IPVH85R, Millipore) at 125 mA for 95 min. Finally, the membrane was blocked with 5% protease-free Albumin fraction V (catnr. 1ET5.4, Carl Roth GmbH & Co. Kg, Karlsruhe, Germany) for 1 h prior to incubation with the following primary antibodies overnight at +4 °C:

Anti-LAMA3 (1:500, catnr. AMAb91123, Atlas antibodies, Bromma, Sweden)

Anti-LAMB3 (1:5000, catnr. Ab150385, ABCAM, Cambridge, UK)

Anti-LAMB3 (1:2000, catnr. AMAb91161, Atlas antibodies)

Anti-LAMC2 (1:1000, catnr. Ab210959, ABCAM)

Anti-LAMC2 (1:2000, catnr. AMAb91098, Atlas antibodies)

Anti-claudin-5 (1:5000, catnr. MA5-32614, Thermo Scientific)

Anti-β-tubulin (1:2000, catnr. 05-661, Millipore)

After incubation, the membrane was thoroughly washed and incubated with HRP-conjugated goat anti-rabbit IgGs (1:2000, catnr. 7074S, Cell Signaling Technology, Danvers, MA, USA) or HRP-linked horse anti-mouse IgGs (1:2000, catnr. 7076S, Cell Signaling Technology) for 45 min at RT. After another thorough wash, Immobilon Chemiluminescent HRP substrate (catnr. WBKLS0500, Millipore) was added to the membrane and the signal density was measured using ODYSSEY FC imaging system (LI-COR Biosciences, Lincoln, NE, USA). The membrane was then probed for β-tubulin using mouse anti-human β-tubulin clone AA2 antibodies (1:2000, catnr. 05-661, Millipore) for 2 h at RT. After a thorough wash, the membrane was incubated with HRP-linked horse anti-mouse IgGs (1:2000, catnr. 7076S, Cell Signaling Technology) for 45 min at RT. Finally, the membrane was incubated with HRP substrate for 30 s and the signal intensity was measured. The images were analyzed with Image Studio (LI-COR Biosciences).

### 4.5. Migration Assay

Use of human blood for this study was approved by the Regional Ethical Review board in Uppsala, Sweden (Dnr. 2015/543). The procedure was performed as described previously [41]. In brief, blood from healthy donors was used to isolate peripheral blood mononuclear cells (PBMCs) using lymphoprep (catnr. 07851, STEMCELL Technologies, Vancouver, BC, Canada). Red blood cells were lysed with Red Blood Cell Lysing Buffer Hybri-Max (catnr. R7757, Sigma-Aldrich) and EasySep^TM^ Human Monocyte Isolation Kit (catnr. 19359, STEMCELL Technologies) was used to isolate untouched monocytes. The cells were stained with BCECF-AsM (catnr. 216254, Sigma-Aldrich) for 1 h at +4 °C. After a thorough wash, 50,000 cells, reconstituted in 200 μL Vasculife medium, were transferred to the upper chambers of a trans-well system with 5 μm pore size (catnr. 9325012, SABEU GmbH & Co. Kg, Northeim, Germany). In the lower chambers, 750 μL conditioned medium from HUVECs cultured on LN332, LN511, or plastic for 48 h were added. Vasculife medium with or without recombinant CXCL12 (80 ng/mL) (catnr. 350-NS-010/CF, R&D systems) in the lower chambers was used as a positive and negative control, respectively. The monocytes were left to migrate for 2 h at 37 °C and 5% CO_2_. The inserts were discarded, and the migrated cells in the lower chambers were left to settle down for 15 min in the incubator prior to capturing five images from five predetermined spots (2.7 mm^2^/spot) within each well. The number of cells in five spots was determined by manual counting.

### 4.6. OLINK Proteomics and Ingenuity Pathway Analysis (IPA)

Supernatants from HUVECs (stored at −80 °C until analysis) were analyzed using OLINK proteomics’ inflammation panel (OLINK Bioscience AB, Uppsala, Sweden). OLINK utilizes proximity extension assay where two antibodies, tagged with complementary nucleotide sequences, bind different epitopes of the same protein. Upon the binding of these antibodies to their epitopes, the complementary nucleotide sequences hybridize and is amplified by PCR. The amplified PCR product is proportional to the amount of the protein and is reported as normalized protein expression (NPX) on a log2 scale.

The paired *t*-test followed by the Benjamini–Hochberg correction for multiple testing was performed on NPX values of proteins released by ECs cultured on an uncoated surface versus LN332. Proteins having a fold-change above 1.5 and FDR of 5% were considered differentially expressed. Enrichment analysis was performed using Ingenuity Pathway Analysis (IPA^®^, QIAGEN Inc., Hilden, Germany. https://www.qiagenbioinformatics.com/products/ingenuity-pathway-analysis, analysis date 14 March 2022) online tool. For this analysis, a cutoff value of 1.5 in protein level and FDR value of 20% for the core analyses were employed to identify enriched functions. Functions with a z-score above 2 or below −2 were considered enriched. The IPA analyses was performed on 14 March 2022.

### 4.7. Adhesion Assay

Twenty-four-well culture plates were coated with 0.5 μg/cm^2^ LN332, LN511, or left uncoated for 2 h at 37 °C. HUVECs (50,000 cells/well) were seeded and incubated for 48 h at 37 °C. HUVECs treated with TNF (50 ng/mL) for 16 h served as positive control. PBMCs were labeled with BCECF-AM (Sigma-Aldrich) for 1 h at +4 °C. Labeled PBMCs were washed twice with PBS supplemented with 2% heat inactivated FBS and 1 mM EDTA and resuspended in Vasculife medium to a concentration of 5 × 10^5^ cells/mL. HUVECs were washed with prewarmed Vasculife and 500 μL (250,000 cells) of prelabeled PBMCs were transferred to each well and incubated for 1 h at 37 °C. The medium and the unbound cells were discarded, and the endothelial monolayer was washed twice with prewarmed Vasculife. Fluorescence intensity in the wells was measured at 485_Ex_/528_Em_ nm and representative images were captured using Cytation 3 plate reader.

### 4.8. Immunocytochemistry

Eight-well chamber slides (catnr. 80841, Ibidi, Gräfelfing, Germany) were coated with LN332, LN511, or left uncoated for 2 h at 37 °C. In total, 3 × 10^4^ HUVECs per chamber were seeded and incubated for 48 h at 37 °C. The cells were washed with ice-cold PBS prior to fixation with 4% paraformaldehyde for 40 min at room temperature. The cells were washed with ice cold PBS and incubated with 1% BSA in 0.1% triton-X100 solution for 30 min at room temperature. They were then incubated with anti-VE-cadherin antibody (1:150, catnr. ab33168, Abcam) for 1 h at room temperature, thoroughly washed, and incubated with Alexa Flour647-conjugated secondary antibody (1:1000, catnr. ab15005, Abcam) for 1 h at RT and in dark. The cells were washed and stained with DAPI for 5 min. After another wash, the slides were air-dried and mounted in PERTEX (catnr. 00811, Histolab, Sweden) and analyzed using Leica TCS SP8X confocal microscopy (Leica, Wetzlar, Germany).

### 4.9. Human Carotid Atheroma Gene Expression Data

Human carotid atheroma gene expression data (GSE43292) obtained from the Gene Expression Omnibus was utilized to evaluate gene expression data from human atherosclerotic tissues. This dataset had previously been described by Ayari and Bricca [48]. In brief, atherosclerotic plaques from 32 hypertensive patients undergoing carotid endarterectomy at Lyon University Hospital were dissected into macroscopically intact (stage I–II) or atherosclerotic diseased tissues (stage IV or higher), according to Stary’s classification of atherosclerotic plaques [49]. The mRNA from these sections was extracted and gene expression was analyzed using Affymetrix Human GeneChip Gene 1.0 ST Arrays. The data were analyzed using the GEO2R web tool.

### 4.10. Statistics

One-sample *t*-tests were used to compare fold change between two groups while a one-way ANOVA and a Bonferroni test were employed to compare multiple groups. The data represent at least three independent experiments unless otherwise indicated. For the analysis of OLINK and microarray data, a *t*-test followed by the Benjamini–Hochberg correction for multiple testing were performed to evaluate statistical significance. Pearson’s correlation was used to evaluate correlation between genes in plaque tissues. *p*-values smaller than 0.05 are considered statistically significant. Data were analyzed in Prism version 9 (GraphPad Software Inc., San Diego, CA, USA).

## Data Availability

The human carotid plaque data are accessible at National Center for Biotechnology Information’s webpage under the accession number GSE43292, https://www.ncbi.nlm.nih.gov/geo/geo2r/?acc=GSE43292 (accessed on 23 April 2023). Additional original data can be provided upon reasonable request.

## Supplementary Materials

The following supporting information can be downloaded at: https://www.mdpi.com/article/10.3390/ijms25168699/s1.
