# Supplementary material for: TNF Induces Laminin-332-Encoding Genes in Endothelial Cells and Laminin-332 Promotes an Atherogenic Endothelial Phenotype
Source: Int J Mol Sci. 2024 Aug 9;25(16):8699. doi: 10.3390/ijms25168699 (PMC11354388; doi:10.3390/ijms25168699)
Supplement: Supplementary file 1 [file ijms-25-08699-s001.zip › ijms-3122471-supplementary.pdf]

Supplementary

TNF induces laminin-332-encoding genes in endothelial cells and laminin-332 promotes an atherogenic endothelial phenotype

Assim Hayderi[1*], Mulugeta M Zegeye[1], Sare Meydan[1], Allan Sirsjö[1], Ashok K Kumawat[1] & Liza U Ljungberg[1]

[1]Cardiovascular Research Centre, Department of Medical Sciences, School of Medicine, Örebro University, Örebro, Sweden

*Corresponding author

assim.hayderi@hotmail.com

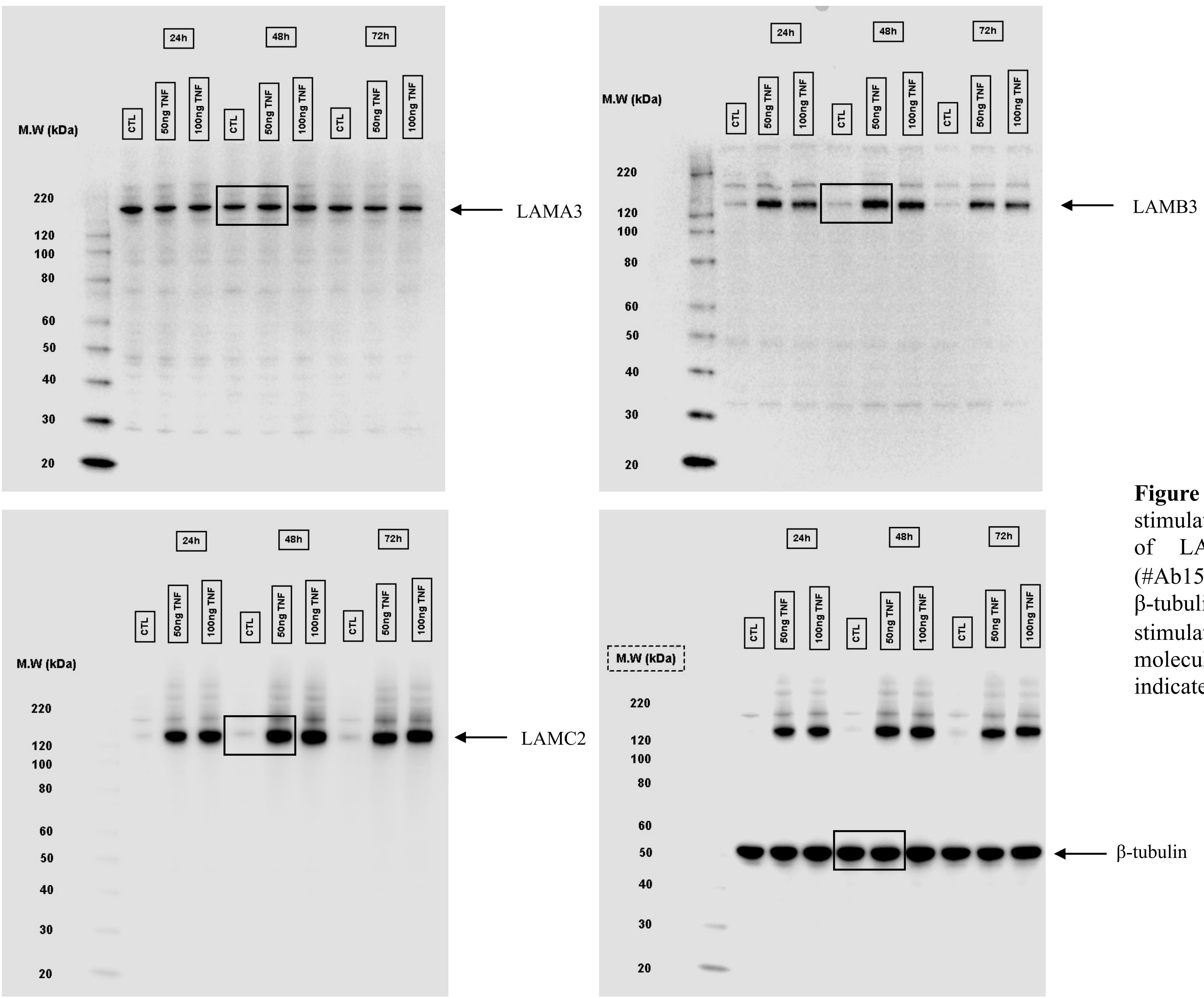

Figure S1. Elevated expression of LN332 subunits in HUVECs stimulated with TNF. Representative full-length Western blot images of LAMA3 (#AMAb91123, Atlas antibodies) (a), LAMB3 (#Ab150385, ABCAM) (b), LAMC2 (#Ab210959, ABCAM) (c) and β-tubulin (#05-661, Millipore) (d) in lysate of HUVECs after stimulation with 50 or 100 ng/ml of TNF for 24-72 hours with molecular weight markers shown in lane 1. The black rectangle indicates the area chosen as representative image in figure 2a.

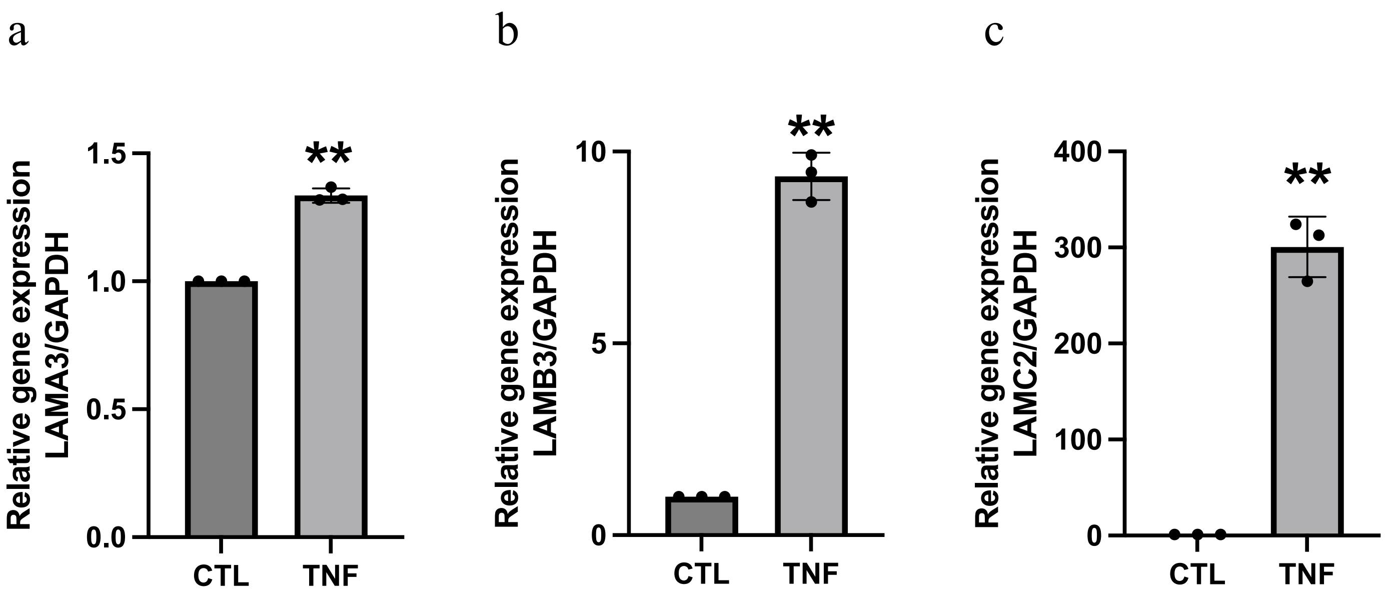

Figure S2. TNF induces LN332-encoding genes in human aortic endothelial cells (HAECs). Expression of *LAMA3* (a), *LAMB3* (b) and *LAMC2* (c) mRNA in HAECs after stimulation with 50 ng/ml TNF for 48 hours. The data are presented as mean±SD of three independent experiments. One sample T-test was performed to assess statistical significance. **P-value <0.01

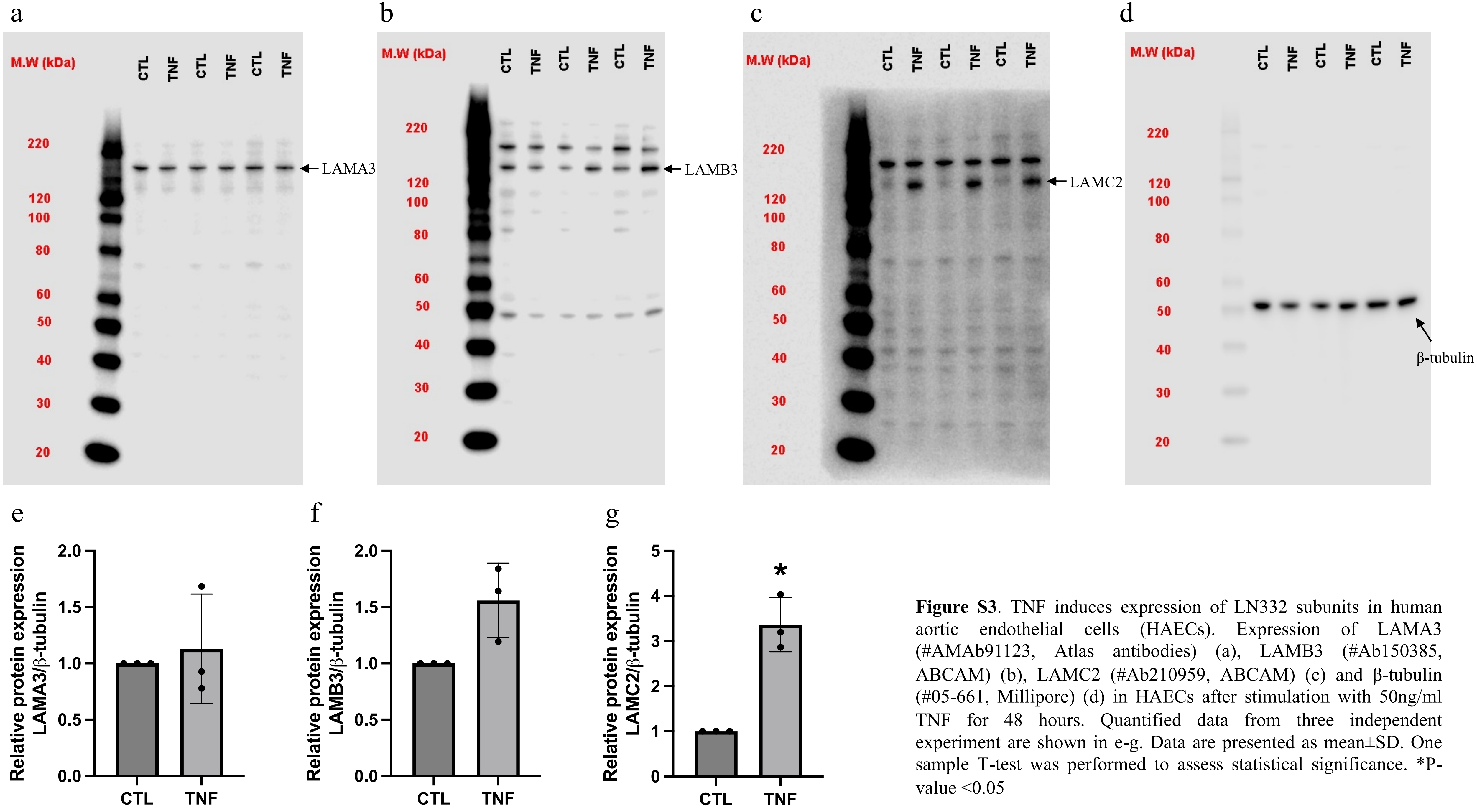

Figure S3. TNF induces expression of LN332 subunits in human aortic endothelial cells (HAECs). Expression of LAMA3 (#AMAb91123, Atlas antibodies) (a), LAMB3 (#Ab150385, ABCAM) (b), LAMC2 (#Ab210959, ABCAM) (c) and β-tubulin (#05-661, Millipore) (d) in HAECs after stimulation with 50ng/ml TNF for 48 hours. Quantified data from three independent experiment are shown in e-g. Data are presented as mean±SD. One sample T-test was performed to assess statistical significance. *P-value <0.05

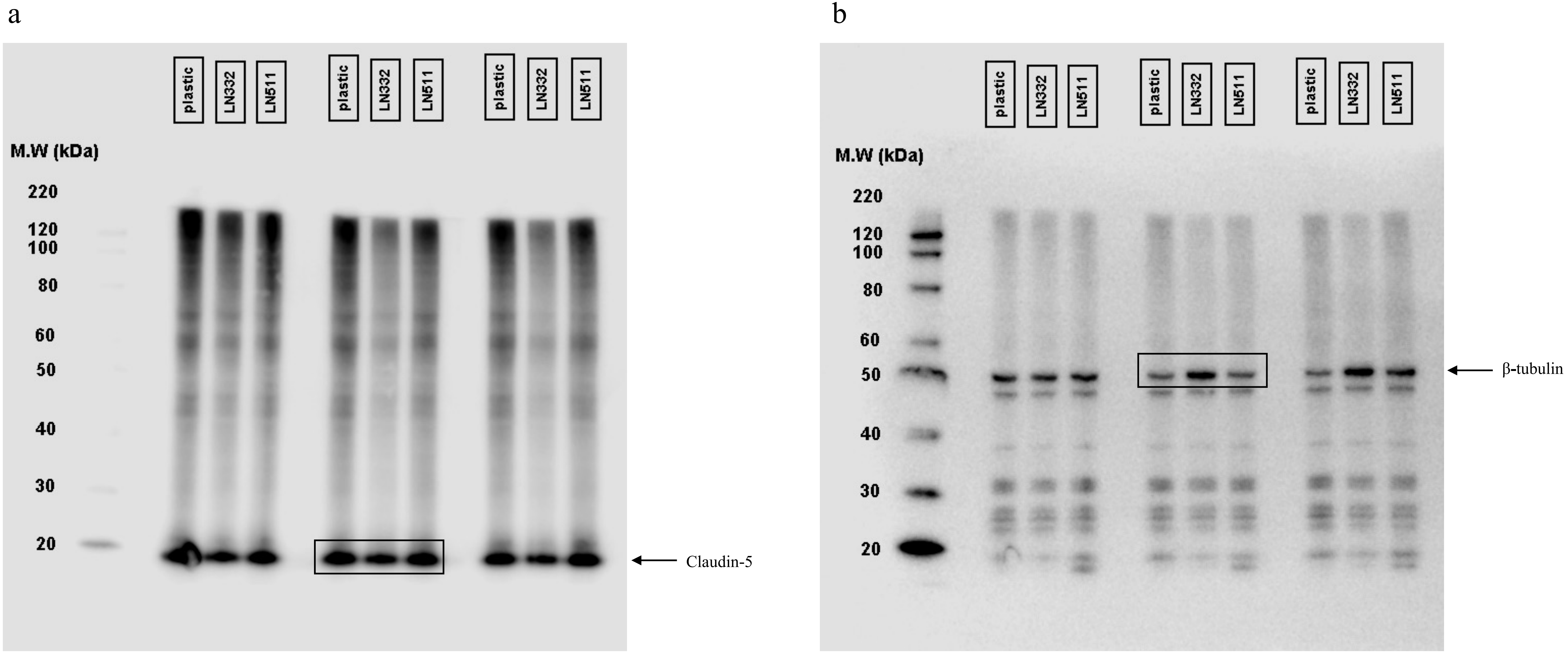

Figure S4. LN332 reduces claudin-5 protein expression in HUVECs. Representative full-length Western blot images of claudin-5 (a) and β-tubulin (b) expression in HUVECs cultured on plastic, LN332 or LN511 for 48 hours (n=3). The black rectangles indicate the areas chosen as representative images in figure 3b. One sample T-test was performed to assess statistical significance. *P-value <0.05

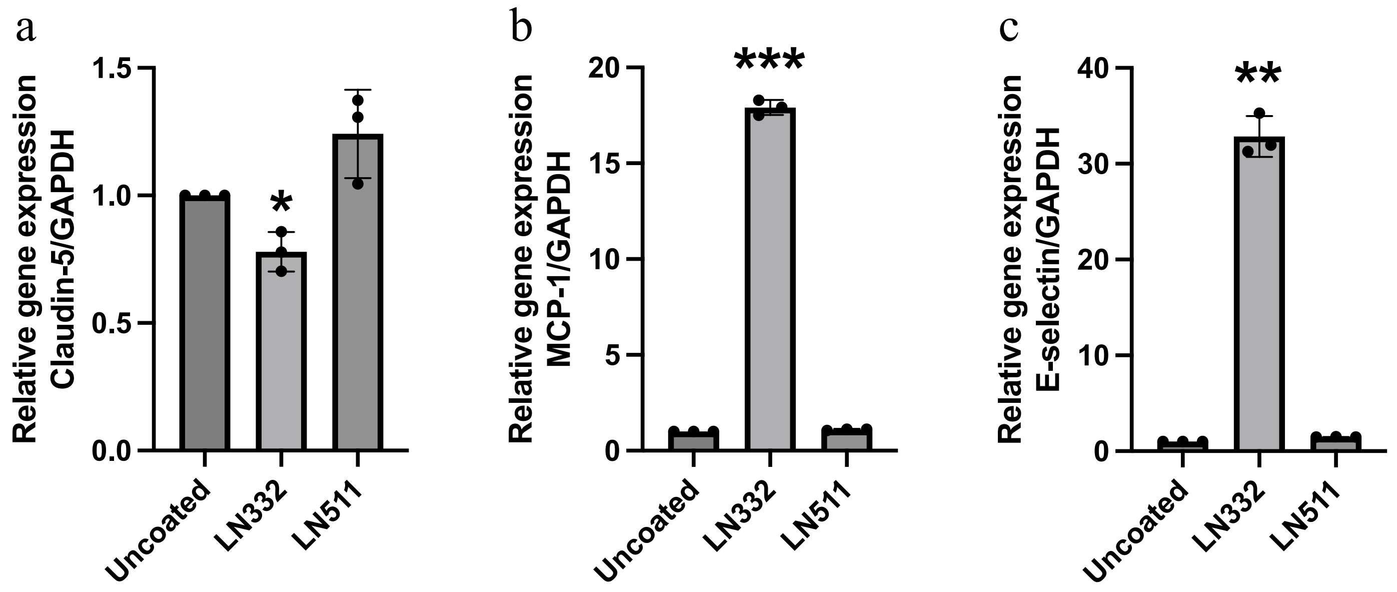

Figure S5. LN332 induces *CCL2 (MCP-1*) and *E-selectin* while it reduces *claudin-5* expression in human aortic endothelilal cells (HAECs). mRNA expression of *claudin-5* (a), *MCP-1* (b) and *E-selectin* (c) in HAECs cultured on uncoated surface, LN332 or LN511 for 48 hours. The data are presented as mean±SD of 3 independent experiments. One sample T-test was performed to assess statistical significance. *P-value <0.05, **P-value <0.01, ***P-value <0.001

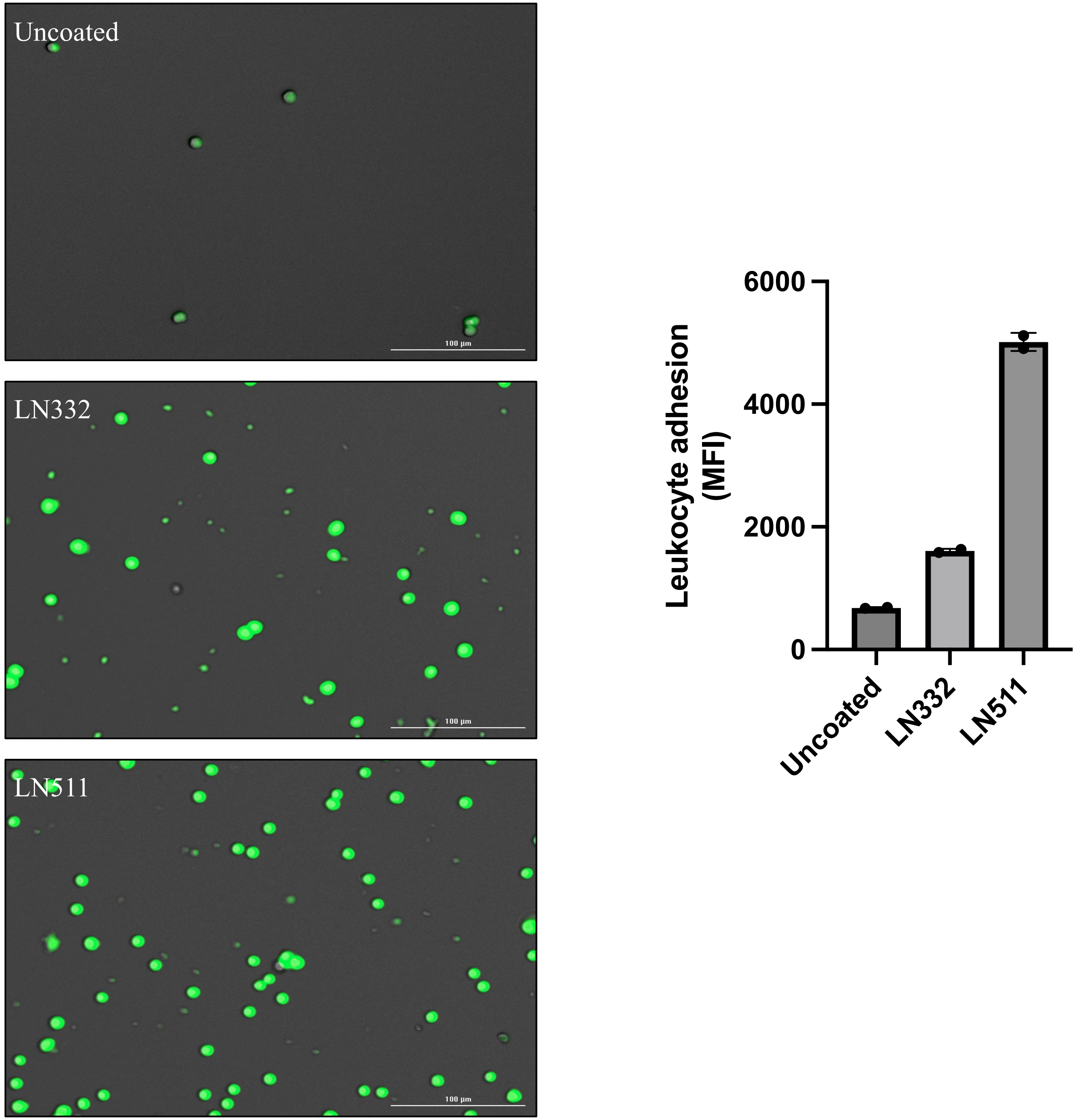

Figure S6. Leukocytes tend to adhere more to LN511 compared to LN332 or uncoated surfaces in the absence of endothelial cells. Leukocyte adhesion to uncoated surface, LN332 and LN511 in the absence of endothelial cells. The data are presented as mean±SD of two independent experiments.
